# Improved Quantum–Classical Treatment of N_2_–N_2_ Inelastic Collisions: Effect of the Potentials and Complete Rate Coefficient Data Sets

**DOI:** 10.1021/acs.jctc.3c01103

**Published:** 2023-11-26

**Authors:** Qizhen Hong, Loriano Storchi, Quanhua Sun, Massimiliano Bartolomei, Fernando Pirani, Cecilia Coletti

**Affiliations:** † State Key Laboratory of High Temperature Gas Dynamics, Institute of Mechanics, Chinese Academy of Sciences, 100190 Beijing, China; ‡ Dipartimento di Farmacia, Università Gabriele d’Annunzio Chieti-Pescara, via dei Vestini, 66100 Chieti, Italy; § School of Engineering Science, University of Chinese Academy of Sciences, 100049 Beijing, China; ∥ Instituto de Física Fundamental—CSIC, C/Serrano 123, 28006 Madrid, Spain; ⊥ Dipartimento di Chimica, Biologia e Biotecnologie, Università di Perugia, via Elce di Sotto 8, 06123 Perugia, Italy

## Abstract

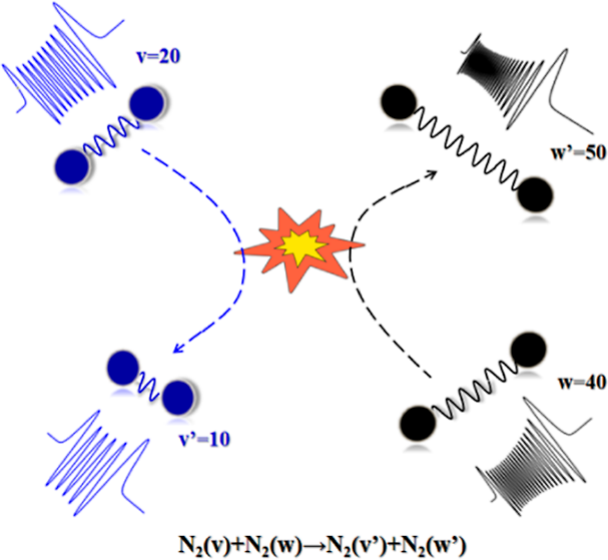

In this study, complete (*i.e.*, including all vibrational quantum numbers in an N_2_ vibrational ladder) data sets of vibration-to-vibration and vibration-to-translation rate coefficients for N_2_–N_2_ collisions are explicitly computed along with transport properties (shear and bulk viscosity, thermal conductivity, and self-diffusion) in the temperature range 100–9000 K. To reach this goal, we improved a mixed quantum–classical (MQC) dynamics approach by lifting the constraint of a Morse treatment of the vibrational wave function and intramolecular potential and permitting the use of more realistic and flexible representations. The new formulation has also allowed us to separately analyze the role of intra- and intermolecular potentials on the calculated rates and properties. *Ab initio* intramolecular potentials are indispensable for highly excited vibrational states, though the Morse potential still gives reasonable values up to *v* = 20. An accurate description of the long-range interaction and the van der Waals well is a requisite for the correct reproduction of qualitative and quantitative rate coefficients, particularly at low temperatures, making physically meaningful analytical representations still the best choice compared to currently available *ab initio* potential energy surfaces. These settings were used to directly compute the MQC rates corresponding to a large number of initial vibrational quantum numbers, and the missing intermediate values were predicted using a machine learning technique (*i.e.*, the Gaussian process regression approach). The obtained values are reliable in the wide temperature range employed and are therefore valuable data for many communities dealing with nonlocal thermal equilibrium conditions in different environments.

## 1 Introduction

State-to-state rate coefficients for vibrational energy transfer processes are needed for the kinetic modeling of nonlocal thermal equilibrium (non-LTE) conditions, which are found in a variety of environments and applications occurring in different temperature ranges and are therefore of interest to many communities. Specifically, cold (and often ultracold) interstellar media and planetary atmospheres are governed by non-LTE conditions. In addition, growing applications involving cold plasma require knowledge of low-temperature cross-sections or rate coefficients for many reactive and inelastic scattering processes. A special case is that of cold air plasmas, mainly composed of nitrogen and oxygen, which are finding increasing use in medicine,^1^ in bioagriculture,^2^ in the production of ammonia (when hydrogen is added) and/or fertilizers as an alternative route with respect to the very unsustainable Bosch–Haber process.^3−6^ On the other hand, hot plasma environments are found in many technological applications ranging from nuclear physics to aerospace science.

The experimental measurement of state-to-state inelastic rates is complicated and requires a precise selection of the initial vibrational states of the colliding molecules as well as the detection of the final states and, depending on the investigated system, is generally limited to the fundamental and low excited vibrational states. Given the applications described above, however, the knowledge of rate coefficients for processes starting from or producing highly excited vibrational states is crucial. The obvious alternative is their theoretical calculation, generally carried out through molecular dynamics techniques combined with accurate electronic potential energy surfaces (PESs). The production of such detailed rates might however be challenging: (i) quantum effects might arise in the nuclear motion, particularly at low collision energies (where some transitions can be classical forbidden events), and they should be included in the dynamical treatment; (ii) the need of rate coefficients spanning a very wide temperature range requires an accurate PES at short, long, and very long interaction distances; (iii) in order to cover the whole vibrational ladder, very many initial vibrational states have to be considered, which means that a huge number of calculations needs to be carried out, so that the computational burden of each calculation should not be too expensive. In the last years, we tackled this problem by using a combined strategy consisting of the application of a mixed quantum–classical (MQC) dynamics method, allowing the quantum description of molecular vibrations and rotational–vibrational coupling, and a classical representation of the remaining degrees of freedom, and an analytical nonreactive PES, built according to the improved Lennard-Jones (ILJ) model.^7^ We were able to produce large tables and data sets of vibration-to-vibration (V–V) and vibration-to-translation/rotation (V–T/R) rate coefficients in a wide temperature range for a variety of systems,^8−13^ covering many initial vibrational states of the colliding dimers, not too close to the dissociation limit. The latter choice is because the MQC method is based on an internally consistent use of a Morse-like intramolecular potential and a corresponding Morse-like vibrational wave function, which are known to be deficient for the representation of highly excited states.

Nevertheless, as remarked by some reviewers, the issue of the highest vibrational states the Morse representation can handle, as well as the ability of the ILJ nonreactive PES to represent effects in the intermolecular interactions arising from the diatoms’ distortion, remains an open question. Up to now, we empirically addressed the matter by comparing the vibrational energy levels of the Morse wave functions to those of the available intramolecular *ab initio* potentials and, for the intermolecular interaction, by monitoring the capability of the ILJ model to correctly reproduce variations in the intermolecular potential due to molecular polarizability and multipole moments upon diatoms’ deformation against *ab initio* calculations. In the present work, we directly face this issue by separately considering the effect of an *ab initio*-based representation of the *intra*molecular potential and the corresponding vibrational wave function versus the Morse ones and the impact of an *ab initio inter*molecular potential versus the ILJ description, on the calculation of V–V and V–T/R rates at increasing initial vibrational quantum states. This will allow us, on the one hand, to explore the validity limit of the above description and, above all, to extend the area of the highly excited vibrational states that can be safely included in the calculations, moving toward the diatom dissociation limit. To achieve this goal, we modified the original MQC method by removing the limitation of the use of Morse potential and wave function. The new code is flexible and can handle any intramolecular potential such as those provided in commonly used full-dimensional PESs.

This new formulation is tested here for the calculations of rate coefficients for V–V and V–T/R energy exchange processes in N_2_–N_2_ collisions. We previously investigated this system^8^ using the standard Morse-based MQC code and an intermolecular ILJ PES by considering initial vibrational N_2_ states up to 40, which we believed to be the highest vibrational state that could be reached preserving a reasonable accuracy. Molecular nitrogen being the main component of earth’s (and other planets’) atmosphere, the determination of data or properties derived for N_2_–N_2_ collisions is of utmost interest, first of all, precisely for the modeling of such atmospheres. The modeling of cold air plasma, as mentioned before, is equally relevant. The characterization of the population of vibrationally excited molecular nitrogen in plasma discharges and the possibility of modulating the plasma operating conditions in such a way to favor vibrational excitation (vibrational pumping^14^) up to the dissociation limit are important tools for the technological development of plasma-based sources for the production of fertilizers, ammonia or nitrogen-based chemicals.^3−6^ Besides, the postshock gas embedding the surface of hypersonic re-entry or hypersonic cruise aircrafts is a highly non-LTE environment that can reach temperatures of several thousand degrees; therefore, the knowledge of rate coefficients for inelastic and reactive processes occurring at high and very high temperatures becomes essential for the technological development of these objects, as is for other high-temperature plasmas or nitrogen-containing gases in shock tubes.

It is thus no wonder that many PESs have been developed over the years to describe N_2_–N_2_ collisions. PESs based on the fitting of *ab initio* points can usually describe well the reactive channels, *i.e.*, the breaking of one or both nitrogen bonds to give partial or complete dissociation or recombination reactions, and are known to perform well to simulate the dynamics at high or very high temperatures. The most recent ones, to our knowledge, are those of refs (15) and (16), hereafter indicated as UMN and PIPNN, respectively, both based on the same set of nearly 17,000 *ab initio* points calculated at the CASPT2/maug-cc-pVTZ level of theory, with the most recent one considering additional points so to have a total of 21,406 geometries. The most recent PIPNN PES uses a new neural network version of the many-body permutationally invariant polynomial approach adopted in the UMN PES, which should ensure a much higher accuracy.

In the present paper, we compare the results obtained previously^8^ (based on the use of the Morse + ILJ PESs) with those calculated using the newly developed general code on the UMN PES, widely used for dynamical calculations.^15,17^ Besides, as will be detailed in the following, the behavior of the UMN PES is similar to that of the PIPNN PES in some of the relevant regions, and the computational time involved in the calculation (which here, as mentioned above, is a key factor) is slightly more favorable. The purpose of the UMN PES is mainly addressed to calculate the rate coefficients for collision-induced dissociation or recombination processes occurring at high temperatures.^15^ Therefore, the intramolecular part of the potential is expected to be able to correctly describe N_2_ bond deformations up to bond breaking. As far as the intermolecular potential is concerned, this PES should give a very good description at short N_2_–N_2_ distances, whereas the long-range potential, which is very important for the correct simulation of vibrational energy transfer processes, is expected in principle to be less accurate than the ILJ formulation.

The MQC method can also be used to evaluate transport properties within the Wang Chang–Uhlenbeck theory from first principles.^9,18^ The characterization of such properties at high temperatures, where experimental measurements are difficult, is particularly important for the modeling of hypersonic flows and thus for the design of aerospace vehicles, and numerous efforts have been made in the last years for their accurate evaluation.^19,20^ In the present work, we therefore calculate gaseous N_2_ thermal conductivity, shear and bulk viscosity, and self-diffusion coefficient, which were not obtained in our previous work,^8^ and we consider how intra- and intermolecular potentials affect their computation.

We addressed the two effects of the intra- and intermolecular potential separately: we first compare V–V, V–T/R rates, and transport properties calculated using the Morse or the UMN intramolecular potential on the same ILJ intermolecular PES, and then, we evaluate the same quantities calculated on the ILJ or the UMN intermolecular PES, using the same UMN intramolecular N_2_ potential. This comparison will allow us to determine the validity conditions of the different PES representations and the best settings for the description of inelastic collisions as well as to produce a data set of N_2_–N_2_ V–V and V–T/R rate coefficients extending the largest vibrational N_2_ quantum numbers to complete the vibrational ladder. It is worth pointing out that the present MQC method cannot treat bond breaking. Therefore, for the highest vibrational quantum numbers (namely, *v* ≥ 45) and at very high temperature, when reactive collisions play a relevant role in the dynamics, the calculated rate coefficient values might be less accurate, though they are expected to have the correct order of magnitude and are mostly intended as indicative. Nevertheless, those data are presumably much more meaningful than rates evaluated by the first-order theories or arbitrary extrapolation procedures, which are often employed in the kinetic modeling of non-LTE environments, as discussed in detail in ref (3) and shown in ref (13) for N_2_ + H_2_ collisions.

Herein, we calculate cross-sections and rate coefficients for the following inelastic processes, *i.e.*, the V–T/R energy exchanges of type
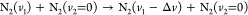
1and
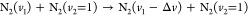
2and the symmetric V–V energy exchanges
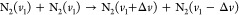
3and the (near-)resonant V–V energy exchanges
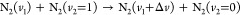
4Note that in the above processes, Δ*v* can be larger than 1, therefore including multiquantum processes, which are very probable at high temperatures and play an important role in the modeling of non-LTE conditions. With the exception of the symmetric process 3, we consider one of the N_2_ molecules in either the ground or first excited vibrational state, which are assumed to be the most populated ones even at a high temperature. The influence of considering higher vibrational states has been shown to be very small for inelastic collisions at the temperature investigated here.^17^ For the other N_2_ molecule (and for process 3), the whole vibrational ladder was included in the calculations in the wide temperature interval 100–9000 K to cover applications including the modeling of planets’ atmospheres and interstellar media, cold and hot plasmas, and hypersonic flows. For higher temperatures, relevant for the latter environment, reactive collisions might become important even for low vibrational states. Therefore, though the method is still expected to work well in this case, the obtained values would need to be checked against data obtained when explicitly considering reactivity.

The present methodology allows rather fast calculations of the selected processes; however, as mentioned above, the direct computation of the whole set of processes corresponding to all vibrational states is a formidable task, requiring a large computational time. Because of this, after having directly calculated large tables of selected processes, we employ machine learning (ML) Gaussian process regression (GPR) models similar to that adopted in ref (21) for inelastic CO + CO collisions and tested against other models for N_2_ + H_2_ collisions^13^ to reliably predict the rate coefficients in the considered temperature range for the missing initial vibrational quantum numbers, producing the complete data sets for the above state-to-state processes.

The structure of the paper is the following: in Section 2, we describe in detail the modifications to the MQC code and the settings of the present calculations; in Section 3, we describe the effect of the intramolecular potential formulation on the V–V and V–T/R rate coefficients as well as on transport properties; Section 4 describes the effect of the intermolecular PES on the same quantities; in Section 5, we summarize the sets of rate coefficients for V–V and V–T/R processes, which were directly calculated and briefly discuss their behavior and apply to them the GP model to produce the complete data sets for N_2_–N_2_ collisions. Conclusions and some remarks on prospective work to increase the data accuracy in the proximity of the dissociation limit are given in the final section.

## 2 The Modified MQC Method

The MQC method, also initially indicated as semiclassical,^22,23^ belongs to the family of methods originally introduced and developed by Billing and is used to calculate cross-sections for the vibrational energy exchange processes of atom–diatom and diatom–diatom collisions. The method simultaneously solves the time-dependent Schrödinger equation for the diatom vibrational degree of freedom and rotational–vibrational coupling and the classical Hamilton equations of motion for the other degrees of freedom. The cross-sections can then be used to calculate the V–V and V–T/R rate coefficients and many transport properties. The quantum treatment of vibration and rotational–vibrational coupling is very often sufficient to recover all important quantum effects arising in these processes, leading to results close to full quantum treatments,^24^ whose use, in the case of molecules with heavy atoms, is hampered by the number of internal quantum states. Besides, like full quantum dynamical treatments, the MQC method completely relies on first-principles; *i.e.*, no external empirical or experimental data are needed, nor is the choice of, for instance, the most appropriate binning procedure, like in quasi-classical trajectory calculations. An external full-dimensional electronic PES is, of course, needed to drive the dynamical evolution of the system.

One of the key ingredients for the solution of the equations characterizing the MQC quantum coupled states treatment is the determination of the matrix coupling elements between the diatoms’ vibrational states, depending on the vibrational wave function and on the intramolecular potential (see the following). The original version of the MQC method is based on the internally consistent adoption of a Morse-like intramolecular potential and the corresponding Morse-like vibrational wave function, allowing the calculation of the matrix elements in an analytical, efficient, and compact manner. However, the limitations of the Morse representation for highly excited vibrational states can reflect on the corresponding results. In this paper, the code is modified to consider any kind of intramolecular potential and vibrational wave function so to remove the bias on its application to high *v* quantum numbers.

Specifically, in the MQC method, vibration and rotational–vibrational coupling are treated quantum mechanically by close coupled equations. First of all, the total wave function is expanded in terms of the product of rotationally distorted vibrational wave functions ϕ_*v*_1__(*r*_1_, *t*)ϕ_*v*_2__(*r*_2_, *t*) as follows
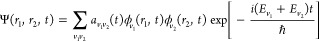
5where  is the expansion coefficient, *r*_*i*_ is the intramolecular distance of diatom *i*, and *E*_*v*_*i*__ is the eigenvalue of the rotationally distorted time-dependent vibrational wave function ϕ_*v*_*i*__(*r*_*i*_, *t*) perturbed by rotational–vibrational coupling
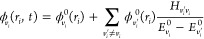
6where  is the first-order centrifugal stretching term
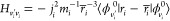
7in which *m*_*i*_ is the reduced mass of the diatom *i*, *j*_*i*_ and  are the classical rotational momentum and equilibrium distance of diatom *i*, respectively, and the operator ⟨ ⟩ is obtained by integrating over *r*_*i*_. Moreover,  and  are the discrete eigenvalues and the associated eigenfunctions of the radial one-dimensional Schrödinger equation
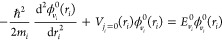
8in which  is the rotationless electronic potential (*i.e.*, the intramolecular potential) of the diatom. In the previous MQC calculations,^8−11,22,23^ the diatomic intramolecular potential was described by the empirical Morse potential, so the analytical solutions (eigenvalues and eigenfunctions) of eq 8 can be obtained analytically and were directly adopted in the calculation. In this paper, to consider any kind of diatomic intramolecular potential, eq 8 is solved numerically by the subroutine SCHRQ of the well-known program LEVEL developed by Le Roy.^25^ Therefore, the eigenvalues, eigenfunctions, and also matrix coupling elements (see the following) can be determined accurately. Note that this calculation is performed once and for all at the beginning of the MQC calculation so that the increase in the computational time with respect to the original code is negligible.

For the expansion coefficients  [amplitudes for the inelastic processes N_2_(*v*_1_) + N_2_(*v*_2_) → N_2_(*v*_1_′) + N_2_(*v*_2_^′^) in this paper], we plug the expansion 5 into the time-dependent Schrödinger equation and obtain the following set of coupled equations
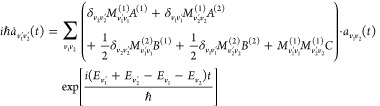
9where
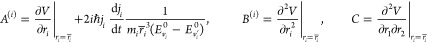
10in which *V* is the intermolecular potential of the colliding system.
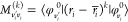
11is called the matrix coupling element and is obtained by numerical integration based on the eigenfunction  of eq 8. Note that in the original MQC calculations,^8−11,22,23^ is integrated analytically using the Morse wave function.

The rotational motion of the diatoms and the relative translational motion are solved by the classical Hamilton equations, making use of an Ehrenfest averaged potential^24^ defined as the quantum expectation value of the interaction potential (see the refs (11) and (24), for details). This mean-field method can provide accurate quantum transition probabilities and properly conserves the total (quantum plus classical) energy. The total wave function, eq 5, is initialized as the product of the eigenfunctions for the two infinitely separated diatoms, namely . The classical Hamilton equations and the coupled eqs (eq 9) are solved by a variable-order variable-step Adams predictor–corrector integrator.^26^ An absolute integration accuracy of 1 × 10^–7^ is achieved for all calculations in this work. Once the quantum transition amplitudes  have been determined, one can obtain the average cross-section (by averaging over a number of trajectories having randomly selected initial conditions) for the vibrational transition as
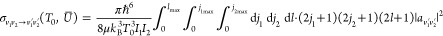
12where μ is the reduced mass for the relative motion of the diatoms and *l* is the orbital angular momentum. The moment of inertia is *I*_*i*_ = *m*_*i*_*r*_*i*_^2^, and the temperature *T*_0_ is arbitrary because it cancels when calculating the rate coefficients. *j*_1max_ and *j*_2max_ are the upper limit for the randomly chosen rotational quantum numbers for the diatoms, and *l*_max_ is the upper limit for the angular momentum. The rate coefficients for vibrational energy exchange can now be calculated through the following equation
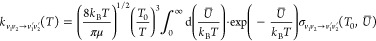
13which holds for the exothermic process, and the symmetrized classical energy *U̅* is introduced to restore detailed balance principle.^22,24^

The MQC calculations in this paper were carried out by running trajectories at 47 initial values of total classical energy comprised between 35 and 80,000 cm^–1^, with a more frequent sampling directed toward lower energies. For each classical energy value, 2000 trajectories (a smaller number than those needed by the quasi-classical trajectory (QCT) method to reach convergence since the MQC method has two quantum degrees of freedom) were considered, which should ensure accuracy for rate coefficients of *ca.* 15%. An initial separation of the diatoms equals 15 Å for V–V and 50 Å for V–T/R energy transfer, and an impact parameter randomly chosen between 0 and 9 Å have been employed. The trajectories were terminated at the same values of the initial separation distances. Much larger initial and final separation distances are needed for the calculation of V–T/R rates, particularly at low temperature when transition probabilities are small, to avoid artificial boundary effects arising in the evaluation of the *A*^(*i*)^ term in eq 10, where the very long-range interaction terms involving multipoles can still give a significant contribution.^24^ For energy transfer processes with different initial vibrational states (*v*_1_*v*_2_), we included in the coupled eqs (eq 9) at least 49 states in the neighborhood of (*v*_1_*v*_2_), and for high values of (*v*_1_*v*_2_), additional states were considered until convergence was reached.

In addition to vibrational inelastic cross-sections and rate coefficients, transport properties (including shear and bulk viscosities, thermal conductivity, and self-diffusion coefficient) for diatomic gases can be calculated through the present MQC method,^18^ combining it with the first-order Sonine expansion of the Wang Chang–Uhlenbeck theory^27,28^ (which takes inelastic collisions into consideration). Specifically, the shear viscosity η, self-diffusion coefficient *D*, and thermal conductivity λ are defined, respectively, as
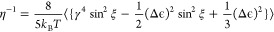
14
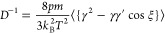
15and
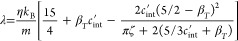
16
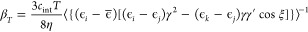
17where ξ is the scattering angle, γ^2^ = μ*v*^2^/2*k*_B_*T* and γ′^2^ are the precollision and postcollision reduced energy of the colliding pair,respectively, *p* is the pressure of the gas, and *m* is the mass of diatom. *c*_int_′ = *c*_int_/*k*_B_, and *c*_int_ is the internal heat capacity. Indices *i* and *j* denote precollision internal states of the colliding pair with the internal energy of diatom being *E*_*i*_ and *E*_*j*_, and *k* and *l* are the postcollision internal states. ϵ_*i*_ = *E*_*i*_/*k*_B_*T* and Δϵ = ϵ_*k*_ + ϵ_*l*_ – ϵ_*i*_ – ϵ_*j*_. ζ is the rotational relaxation collision number defined by^29^
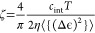
18Furthermore, the bulk viscosity η_*V*_ is given by^29^
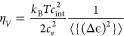
19in which *c*_*v*_ is the thermal capacity at constant volume.

A Monte Carlo procedure is used to average over the molecular collisions, computed by the present MQC method, to obtain the collision integrals ⟨{}⟩ (appearing in the above formulas)
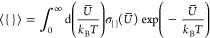
20with
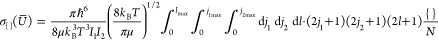
21where *N* is the number of trajectories calculated at a given value of the symmetrized classical energy *U̅*. For such calculations, both N_2_ molecules were considered in the initial ground vibrational state, and the remaining settings are the same as for calculating the V–V cross-sections.

## 3 Effect of Intramolecular Potential on V–V and V–T/R Rates and on Transport Properties

In this section, we evaluate the performance of the UMN and the Morse potentials for molecular nitrogen by calculating selected V–T/R [namely, process (1) with Δ*v* = 1] and V–V quasi-resonant processes (3) and (4) rate coefficients as well as shear and bulk viscosities, thermal conductivity, and self-diffusion coefficients in the whole temperature range considered here. Because in the above processes the initial quantum number *v*_2_ is fixed to 0 or 1 or is the same as *v*_1_, in the following, we simplify the notation by using *v* = *v*_1_.

**Figure 1:**
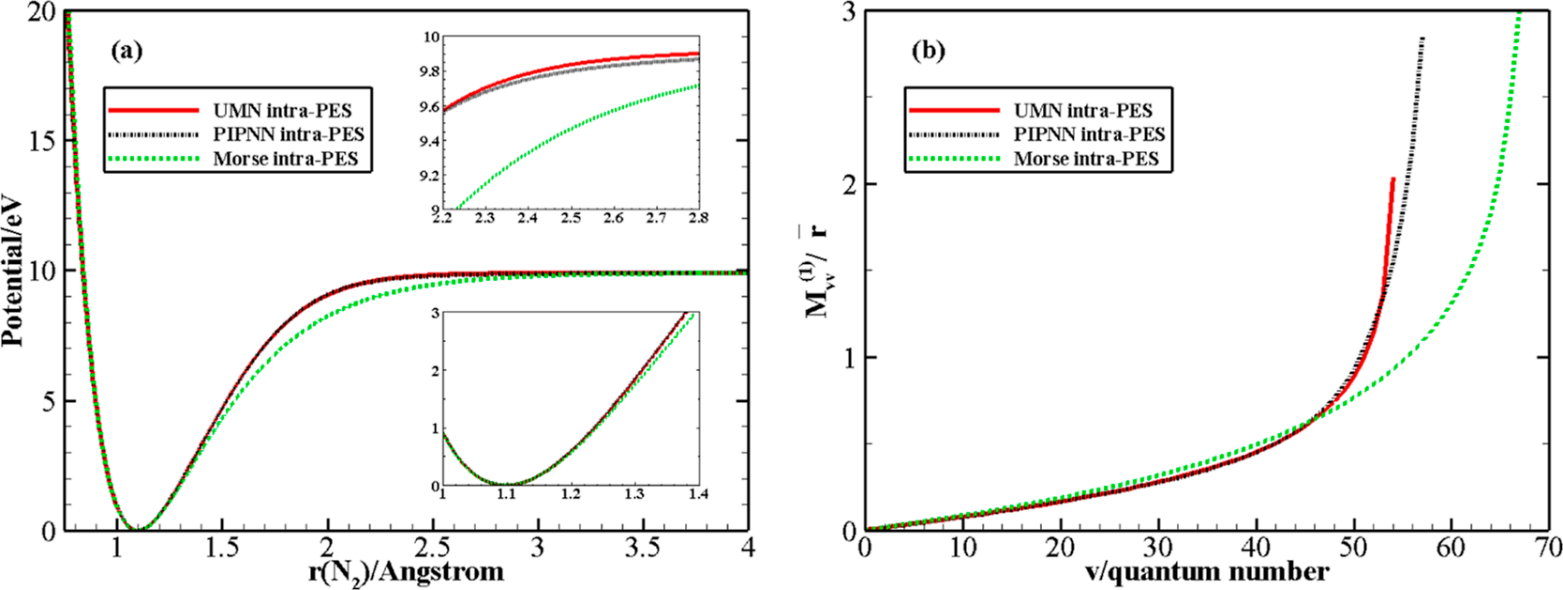
(a) Behavior of the ILJ, UMN, and PIPNN intramolecular potentials as a function of the intramolecular distance of N_2_. (b) Coupling matrix elements  (eq 11) divided by N_2_ equilibrium distance *r̅*.

The UMN and Morse potentials for the N_2_ molecule are compared in Figure 1, panel a (Table S1 in the Supporting Information reports the employed Morse parameters). The corresponding vibrational energy levels obtained with the LEVEL code^25^ are reported in Table S2 in the Supporting Information. They amount to 68 for the Morse potential and 55 for the UMN PES. It is worth remarking that there is no consensus over the total number of vibrational states, and different *ab initio* PESs can account for different numbers. For instance, the PIPNN PES, though similar to the UMN PES (Figure 1), leads to 58 vibrational states. As was expected, the Morse and UMN intramolecular PESs closely agree at the bottom of the well, *i.e.*, for not too large deviation from the equilibrium distance, with the Morse potential rising more gradually for internuclear distances larger than 1.5 Å. Panel (b) of Figure 1 shows the coupling matrix elements  (eq 11): the values obtained by UMN, PIPNN, and Morse potentials match rather well up to *v* = 20; the agreement is still good up to *v* = 44 (within 10%) with the difference growing rapidly for higher *v* values. In addition, good agreement between the UMN and PIPNN intramolecular PESs is found except for the near-dissociation limit.

**Figure 2:**
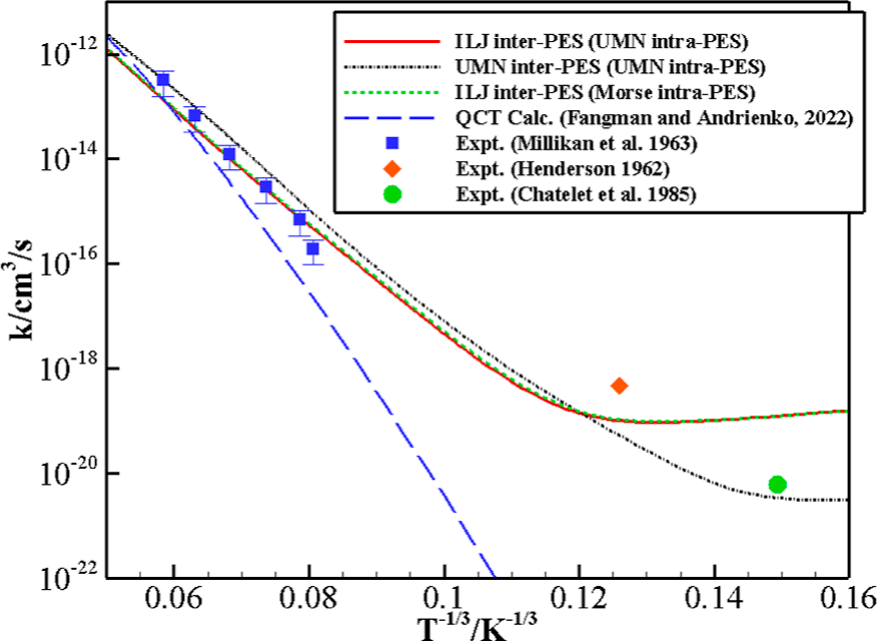
Landau–Teller plot of the rate coefficients for the transition (1, 0) → (0, 0) on the ILJ intermolecular PES with UMN (full red line) and Morse (dashed green line) intramolecular PESs. The dash–dot black line denotes the results based on the UMN intermolecular and intramolecular PESs. Experimental data of ref (30) (blue squares), ref (31) (red diamonds), and ref (32) (green circle) and the QCT calculations^17^ (long-dashed blue line) based on the UMN PES are also reported.

**Figure 3:**
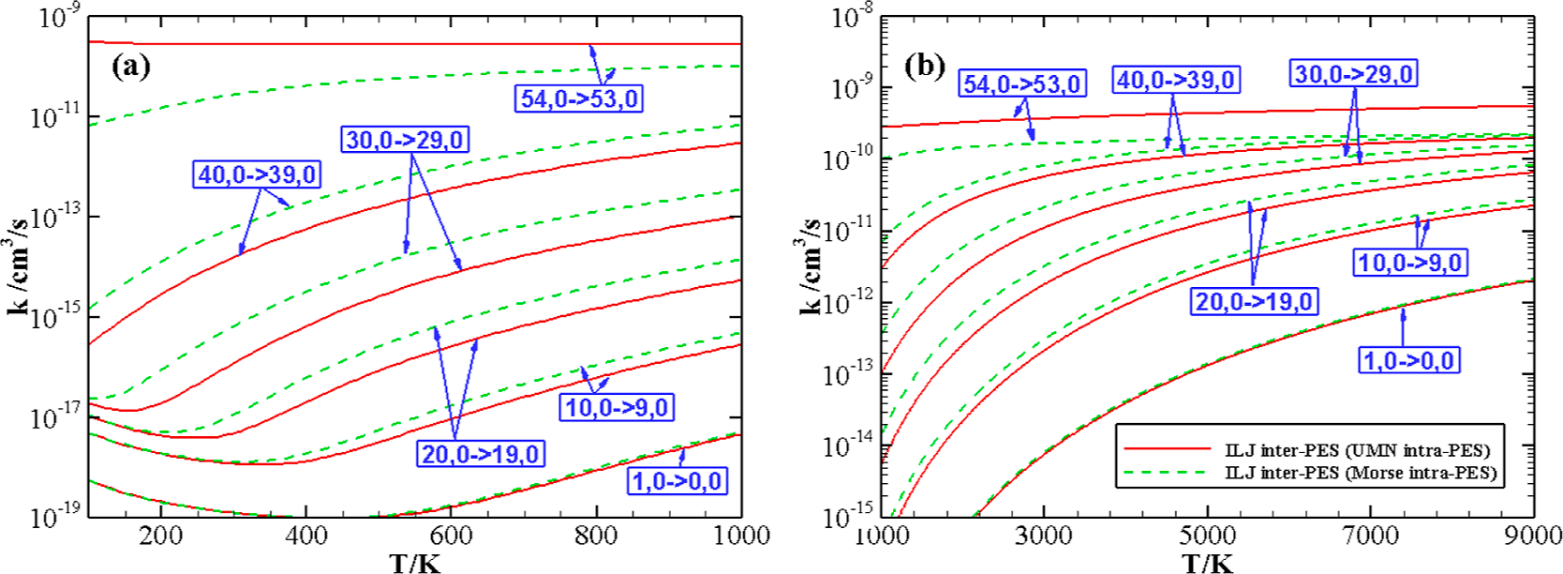
V–T/R rate coefficients as a function of temperature (100–1000 K, panel a, and 1000–9000 K, panel b) for the (*v*, 0) → (*v* – 1, 0) transitions calculated on the ILJ intermolecular PES with UMN (full red line) and Morse (dashed green line) intramolecular PESs.

Figures 2 and 3 show the comparison between the V–T/R rate coefficients calculated with the vibrational energy levels and coupling matrix elements based on the Morse and on the UMN intramolecular PESs. Figure 2 reports the calculated V–T/R rate coefficients as a function of temperature for the (1, 0) → (0, 0) transition together with the available experimental data and the rates^17^ derived from the curve fits (valid in the temperature range 5000–30,000 K) of the QCT calculations on the UMN PES, and Figure 3 compares the rates for the (*v*, 0) → (*v* – 1, 0) transitions at *v* = 1, 10, 20, 30, 40 and the highest 54 quantum numbers, focusing on different temperature ranges, (100–1000 K), panel a, and (1000–9000 K), panel b.

As expected, the rate coefficients for (1, 0) → (0, 0) process calculated with the Morse and UMN intramolecular potential hardly show any difference, and both produce values that are in excellent agreement with the measured data, as found in our previous work.^8^ However, as *v* grows, the rates become gradually different: for *v* up to 20, the results lie within a factor of 2. It is worth noting that the differences are small at low temperature as well as at very high temperature. For all the V–T/R coefficients within *v* = 20, a non-Arrhenius behavior of the rates can be observed upon the use of both Morse and UMN intramolecular potentials, with a small change of the transition temperature (from the Arrhenius to the anti-Arrhenius behavior) increasing with *v*.

For *v* = 30 and *v* = 40, the difference becomes significant and is larger than the accuracy of the method, rising to a factor of 3 or 4 at most. One can again note that the difference reduces at higher temperatures. The discrepancies in the whole temperature range are however more reliable than those obtained by the first-order theory or by widely employed extrapolation techniques, which might present orders of magnitude differences with the calculated data (see, *e.g.*, ref (13)). For the highest *v* = 54, the rate coefficients are very different both at low (up to 2 orders of magnitude) and high (a factor of 3) temperatures. In this case, however, the UMN and Morse energy levels (and the corresponding energy mismatches) are so dissimilar that a direct comparison might lose physical meaning.

**Figure 4:**
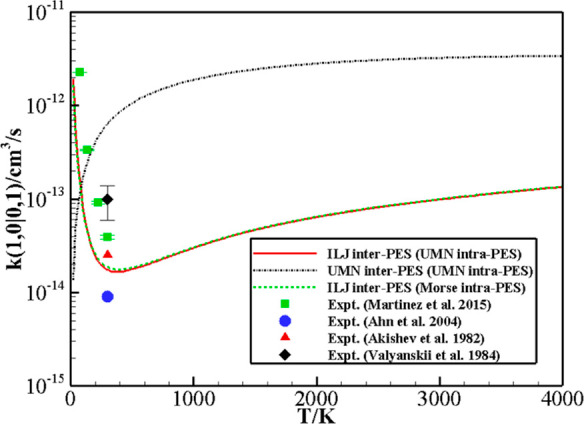
Rate coefficients as a function of temperature for the (1, 0) → (0, 1) transition calculated with the UMN (full red line) and the Morse (dashed green line) intramolecular PESs on the ILJ intermolecular PES. The dashed-dotted black line denotes the results based on the UMN intermolecular and intramolecular PESs. Experimental data of ref (33) (blue circles), ref (34) (black diamonds), ref (35) (green squares), and ref (36) (red triangles) are also reported.

**Figure 5:**
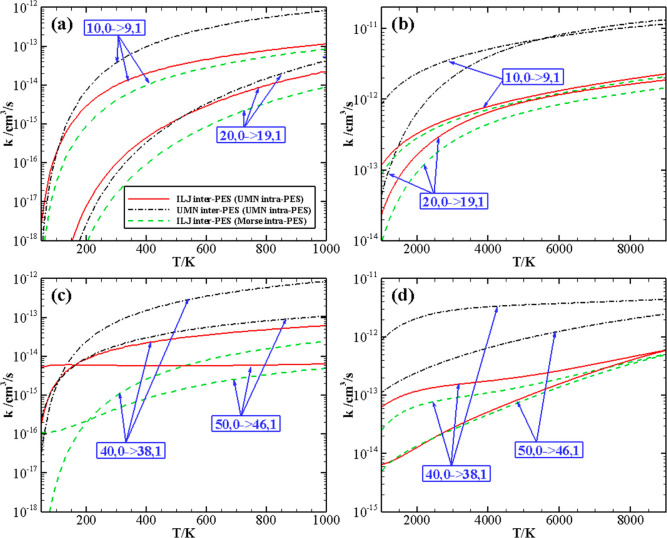
Rate coefficients as a function of temperature (50–1000 K, panels a and c, and 1000–9000 K, panels b and d) for the (*v*, 0) → (*v* – Δ*v*, 1) transition calculated with the UMN (full red line) and the Morse (dashed green line) intramolecular potentials on the ILJ intermolecular PES. The dashed–dotted black line denotes the results based on the UMN intermolecular and intramolecular potentials.

V–V rates calculated with the UMN and the Morse intramolecular PESs on the ILJ intermolecular PES are compared in Figures 4 and 5. Figure 4 reports the comparison of the calculated V–V rate coefficients as a function of temperature for the symmetric (1, 0) → (0, 1) transition and the available experimental data:^33−36^ Only tiny differences are obtained between the rates calculated using the Morse and the UMN intramolecular potentials. Note that they both again give good agreement with the experimental data available at very low temperatures, showing a strong anti-Arrhenius behavior, which the calculated rates are able to fully reproduce. This behavior, as described in the following section, is indeed mainly connected to the shape of the van der Waals intermolecular well. Figure 5 compares the V–V rate coefficients as a function of temperature for symmetric and asymmetric (*v*, 0) → (*v* – Δ*v*, 1) quasi-resonant transitions for growing *v* values up to 50, near the dissociation limit of the UMN intramolecular potential. As shown in panels a and b (along with Figure 4), for the (*v*, 0) → (*v* – 1, 1) processes, the differences between the calculations with the UMN and the Morse intramolecular potentials gradually grow as *v* increases and as the temperature decreases. As a consequence of this behavior, the discrepancies are near the accuracy of the method for *T* ≥ 2000 K (panel b), rising to one (or more) order of magnitude at very low temperature (panel a). At such temperature values, however, the rates are very small; *i.e.*, those processes, in either case, are not expected to play any role in the kinetic modeling of N_2_ gas/plasma.

**Figure 6:**
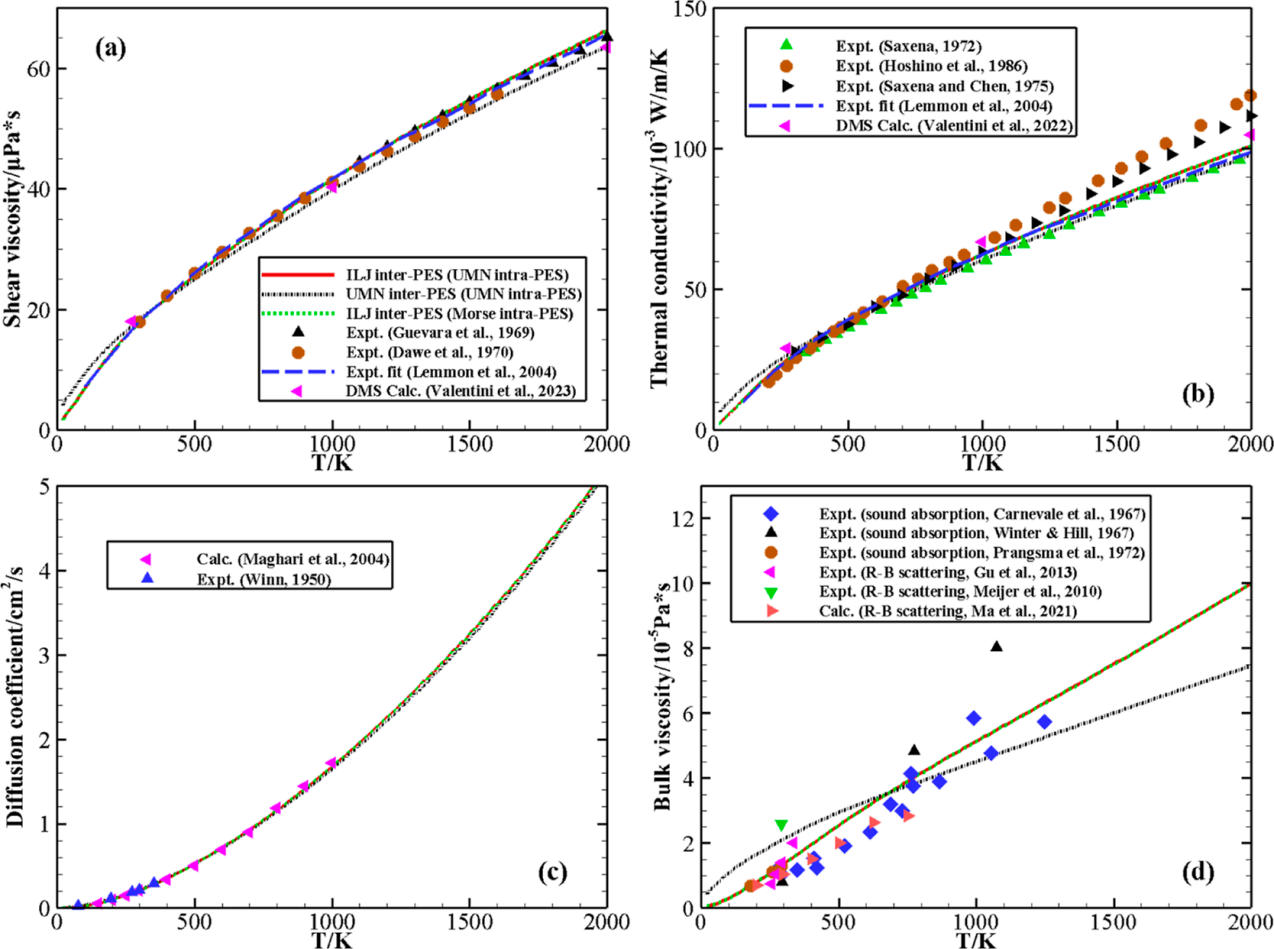
Transport coefficients of N_2_ as a function of temperature. The solid red and dashed green lines denote the calculations based on the ILJ intermolecular with the UMN and Morse intramolecular potentials, respectively, while the black dotted line represents the UMN potential-based results. (a) Shear viscosity. Experimental data of refs (37−39) and the results of direct molecular simulation (DMS)^20^ are also reported. (b) Thermal conductivity. Experimental data of refs (39−42) and the DMS results^19^ are also reported. (c) Self-diffusion coefficient. Experimental data^43^ and the calculated results in ref (44) are also reported. (d) Bulk viscosity. Experimental data of refs (45−49) and the calculated results in ref (50) are also reported.

Panels c and d show the rate coefficients for some exemplary near-resonant processes, which present higher rates at both low and high temperatures: processes (40, 0) → (38, 1) and (50, 0) → (46, 1), with energy deficit Δ*E* (that is the difference between initial and final vibrational energy) of −165.61 (or −357.29) cm^–1^ for the UMN (or Morse) vibrational levels and −73.45 (or 355.76) cm^–1^ for the UMN (or Morse) vibrational levels, respectively. The rate coefficients for the (40, 0) → (38, 1) process calculated with the Morse and UMN intramolecular potentials differ by a factor of 2 at low temperatures and approach each other as temperature increases. For the (50, 0) → (46, 1) process, the results based on the UMN intramolecular potential are 1 order of magnitude larger below 1000 K due to its smaller absolute Δ*E* than the Morse’s. Interestingly, the two results nearly collapse above 2000 K, which most likely arises from the compensation effect of the differences in the vibrational energies and coupling matrix elements.

The use of the more accurate intramolecular potential for highly excited vibrational states therefore becomes crucial for V–V quasi-resonant processes and reveals that they can give significant contributions, even at low temperature, for the determination of the vibrational distribution function (VDF), relevant for the modeling of cold plasmas.

Transport properties of gaseous N_2_ were calculated as a function of temperature using the MQC cross-sections computed on the same ILJ intermolecular PES to examine the effect of Morse and UMN intramolecular potentials. However, since these calculations are based on the initial ground vibrational state only, very small differences are expected, and indeed, the curves for shear viscosity and thermal conductivity are nearly identical (Figure 6, panels a and b). The calculated shear viscosity is in excellent agreement with all experimental data^37−39^ in the considered temperature range. In particular, the values are in practice superimposable with the experimental correlation proposed by Lemmon and Jacobsen,^39^ which fitted the extensive existing experimental measurements and was generally within 5–6% of the actual experimental data, both at low and high temperatures. Available experimental results for thermal conductivity (Figure 6, panel b), though matching at low temperatures, are quite spread at higher temperatures. The values calculated on the ILJ intermolecular PES agree very well at the lowest temperature and closely match the experimental data of refs (39) and (40), being smaller than the experiments of refs (41) and (42), at high *T* values. Note that in the calculation of thermal conductivity, the neglection of higher-order terms in the Wang Chang–Uhlenbeck theory might reflect on the high-temperature values. However, once again, the comparison with the experimental correlation proposed by Lemmon and Jacobsen^39^ is excellent both at low and high temperatures.

Panel c in Figure 6 shows the calculated values of the self-diffusion coefficient at 1 atm. The results obtained by the two intramolecular potentials again coincide and also give a very satisfactory agreement with the experimental data^43^ available at low temperatures. The predicted self-diffusion coefficient of N_2_ reported in ref (44), which is calculated based on the PES obtained by inversion of experimental viscosity and second virial data, is also shown in Figure 6 and agrees well with our results. Panel d in Figure 6 gives the calculated values of the bulk viscosity, and the results obtained by the two intramolecular potentials are close and lie within the experimental data [sound absorption experiments^45−47^ and Rayleigh–Brillouin (R–B) scattering measurements^48,49^] spread. Besides, the calculated results (obtained by fitting direct simulation Monte Carlo calculated R–B scattering spectra to the molecular dynamics spectra) reported by Ma *et al.*(50) also agree well with the present ones around room temperature but become lower at higher temperatures, the reason likely being that their molecular dynamics simulation adopts a rigid-rotor treatment of molecules and an empirical Lennard-Jones potential, while the present MQC calculation treats the vibrational degrees of freedom accurately.

All the above results converge in indicating that for properties involving low-lying vibrational states Morse and UMN intramolecular potentials give close results with reasonable (*i.e.*, within the accuracy of the method) differences. When considering high-lying vibrational states, the discrepancy becomes larger, though still within 1 order of magnitude, and the use of a more reliable *ab initio*-based intramolecular potential is recommended.

## 4 Effect of Intermolecular Potential on V–V and V–T/R Rates and on Transport Properties

As the UMN intramolecular potential produces more reliable results for the whole vibrational ladder, in this section, we will compare the effect of intermolecular potentials on the calculation of vibrational exchange rate coefficients and transport properties using the UMN-based vibrational energies and matrix elements for all of the considered intermolecular PESs.

At variance with the intramolecular potential, for which the inefficiency of the Morse representation for the description of highly excited vibrational states is clear, here, the correct behavior of the involved intermolecular PESs is much less obvious. As mentioned in the Introduction, some hints may be obtained by the way and the scope with which such PESs were built for and by the comparison with high-level *ab initio* results from selected configurations.

**Figure 7:**
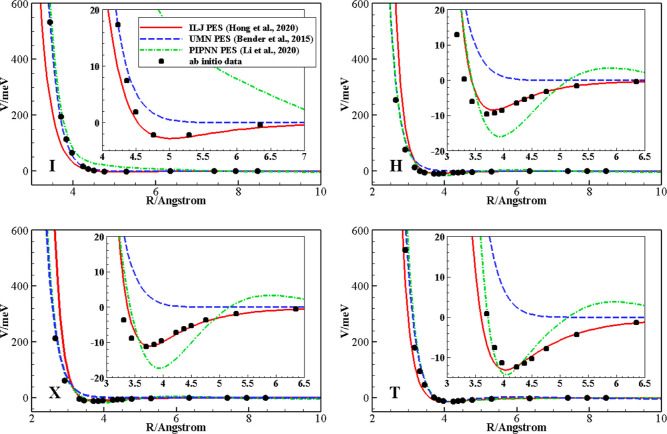
Behavior of the ILJ, UMN, and PIPNN intermolecular PESs as a function of the intermolecular distance for the collinear I (top left), the parallel H (top right), the crossed X (bottom left), and the perpendicular T (bottom right) configurations.

The behaviors of ILJ, UMN, and the most recent PIPNN PESs are therefore shown in Figure 7, together with *ab initio* points calculated at the CCSD(T)/aug-cc-pVQZ plus mid bond function set [3s3p2d1f] level of theory used in ref (8) to validate the ILJ PES, for selected configurations at N_2_ equilibrium distance.

The figure shows that the PESs perform very differently, depending on the intermolecular distance. The ILJ PES matches very well the *ab initio* points at long range, for the description of the van der Waals well and of the first repulsive wall, where instead the UMN and PIPNN PESs show different behaviors: UMN is mostly repulsive, whereas PIPNN often describes well the position of the van der Waals well but not its depth and shows the presence of small barriers moving from the asymptotic region toward the well. In the strong interaction region (at short-range), on the other hand, UMN and PIPNN show a very good agreement with the *ab initio* points, whereas ILJ can be either too attractive or repulsive depending on the selected configuration. The different behaviors reflect the purpose for which the PESs were built. UMN and PIPNN are meant to describe processes occurring at very high temperatures, focusing on reactive collisions; *ab initio* points are therefore gathered in the strong covalent interaction region, and the number of additional geometries required for the description of the van der Waals region with the same accuracy would be enormous to cover the wide long-range interaction area. The ILJ PES, conversely, is based on a phenomenological analytical approach and cannot reproduce covalent interactions arising at short-range; *i.e.*, it is a nonreactive PES, describing in detail the physical interactions arising at medium, long, and very long-ranges.

In the following, comparisons will be made on results obtained by ILJ and UMN PESs only. The reason is that previous calculations on V–V and V–T/R rate coefficients and on transport properties mainly used the UMN intermolecular potential and, above all, because the computational load associated with the more elaborate PIPNN PES, considering a larger number of *ab initio* points, is heavier than the UMN PES. This is an important factor when whole sets of state-to-state quantities need to be determined.

As in the previous section, we compare the values obtained using the two PESs and experimental data, when available, for the same selected V–T/R and V–V processes.

For the (1, 0) → (0, 0) process, V–T/R rates (Figure 2) obtained by the UMN and ILJ PESs are different by a factor of 2 at high temperature and differ by 2 orders of magnitude at the lowest ones. Except for the value measured at the highest temperature, slightly better matched by UMN rates, the ILJ PES gives results in closer agreement with experiments. At low temperature, the very limited experimental data are quite different, preventing an unambiguous comparison with the calculated rates.

**Figure 8:**
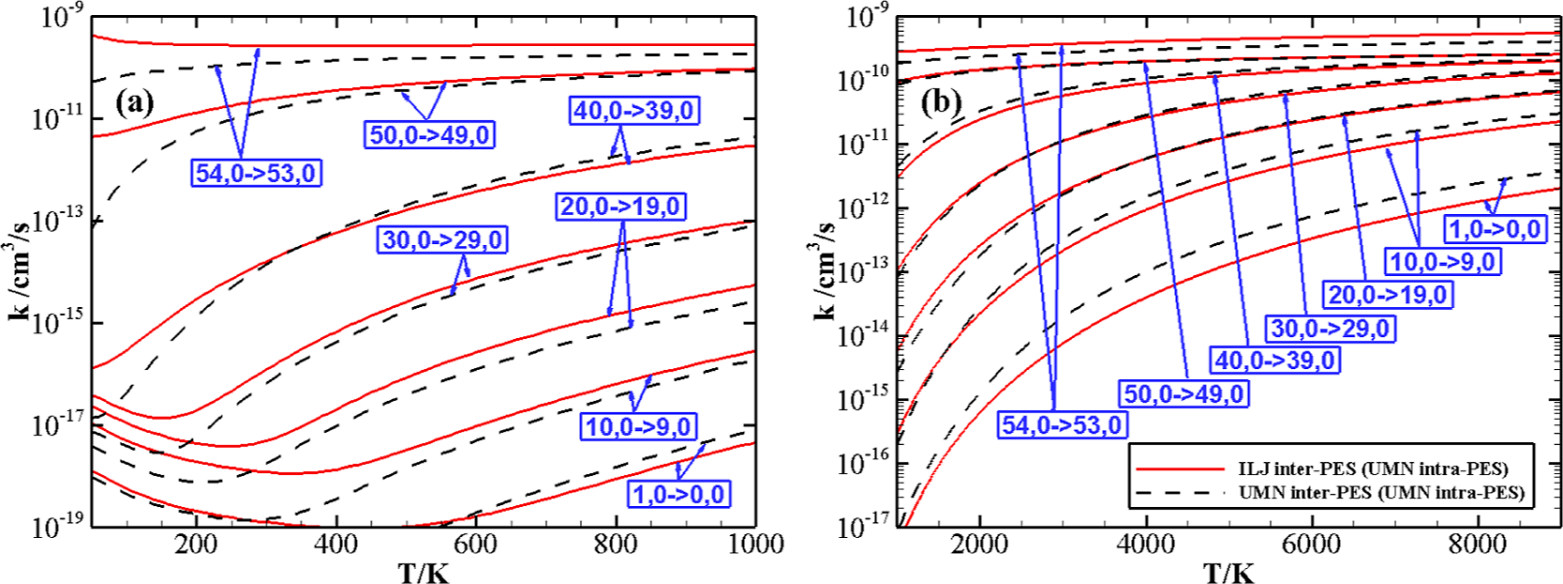
V–T/R rate coefficients as a function of temperature (50–1000 K, panel a, and 1000–9000 K, panel b) for the (*v*, 0) → (*v* – 1, 0) transitions calculated on the ILJ (full red line) and UMN (dashed black line) intermolecular PESs, with vibrational energy levels and matrix elements obtained from the UMN intramolecular PES.

Figure 8 shows the V–T/R rate coefficients for the (*v*, 0) → (*v* – 1, 0) transitions calculated on the ILJ and UMN intermolecular PESs. For temperatures above 300 K, the differences decrease as *v* increases, and they become significant only for *v* = 0 and 10. Below 300 K, on the contrary, the differences are large and amount to 1 or 2 orders of magnitude except for the (1, 0) → (0, 0) process. The use of the two PESs is therefore nearly equivalent in the high-temperature regimes, but the more accurate long-range interaction description of the ILJ PES plays a role at temperatures lower than 300 K.

V–V rate coefficients for the resonant (1, 0) → (0, 1) transition, displayed in Figure 4 together with experimental data, show a remarkable discrepancy between the UMN and ILJ calculated values. The UMN PES completely fails to qualitatively reproduce the anti-Arrhenius behavior found at low *T* by experiments and well described by ILJ PES; UMN and ILJ results quantitatively differ for up to 3 orders of magnitude.

The calculated rates based on the ILJ and the UMN intermolecular PESs are also remarkably different for the symmetric (10, 0) → (9, 1) and (20, 0) → (19, 1) and asymmetric (40, 0) → (38, 1) and (50, 0) → (46, 1) V–V transitions shown in Figure 5. For the symmetric processes, the trend of the UMN and ILJ curves (panels a and b) slightly differs, leading to quantitative discrepancies depending on the temperature and becoming significant at very low and very high *T* values. The disagreement is more relevant for the quasi-resonant processes, particularly at low temperature and tends to level up at the highest temperature investigated here with values within 1 order of magnitude.

V–T/R and V–V results and the nature of the two PESs indicate that the very large difference found at low temperature, where the ILJ PES gives the best match with experimental data, can be due to the description of the van der Waals interaction well and the long-range interaction, which ILJ describes more accurately than UMN. V–T/R rates at high temperature are similar, suggesting that the description of the first repulsive region, which is the main contribution driving the dynamics in such conditions, is good in both cases and not too dissimilar, as indicated by Figure 7. The reason why V–V rate coefficients are instead very different at high temperature is less straightforward to explain: on one hand, the UMN PES better describes the short-range interaction, which plays herein a relevant role; on the other hand, when quasi-resonant processes are investigated, one has to consider that the resonant exchange of one or more quanta of vibrational energy is promoted by the trapping of the colliding molecules in the van der Waals well, better represented by the ILJ PES. Because the similarity of V–T/R results at high *T* suggests that the first repulsive short-range interaction should indeed be similar, the V–V rate difference can be ascribable to the van der Waals well trapping, for which description with ILJ PES is more appropriate.

When it comes to transport properties (Figure 6), based on the ground vibrational state only, the differences upon the use of the two PESs are generally small for shear viscosity, thermal conductivity, and the diffusion coefficient (panels a, b, and c, respectively) and mostly arise at low and high temperatures. Specifically, UMN-calculated values are slightly higher at low and slightly smaller at high *T* values. Comparison with the many available experimental data provides a very good matching, with the ILJ results generally agreeing slightly better, particularly at the lowest temperature, with the recent experimental fit by Lemmon and Jacobsen,^39^ except for thermal conductivity at high temperature. Note that the UMN results are also close to those obtained by direct molecular simulation (DMS),^20^ where the UMN PES was employed. The UMN and ILJ results for the diffusion coefficient are nearly superimposable and show an excellent agreement with both the experiment and the calculations by Maghari and Jalili.^44^

The largest discrepancy is found for bulk viscosity (panel d, Figure 6), for which UMN- and ILJ-based curves show a different trend. The ILJ results are slightly closer to the experimental results (in particular with the most recent ones^48^), which however are rather scattered.

All these data suggest that for nonreactive processes, or whenever reactivity or bond breaking is not involved, the ILJ intermolecular PES provides a better representation of the collisional events both at low and high temperatures; for the latter, the differences with the UMN results are nevertheless smaller. This might as well be the reason for some findings in a recent work by Fangman and Andrienko,^17^ where simulations of N_2_–N_2_ relaxation under postshock conditions were carried out by QCT dynamics with the UMN PES: an excellent agreement with experimental dissociation rate coefficients was found (well described by the UMN PES), whereas some differences arose in the vibrational relaxation.

Furthermore, the analytical representation of the ILJ PES allows us to have a much reduced computational time. As an example, the CPU time involved in the calculations of the same 100 trajectories for the N_2_(*v* = 50) + N_2_(*v* = 0) collision at collision energy of 35 cm^–1^ is 1.2 h upon use of the ILJ PES and 40 h for the UMN PES using a single CPU core. We point out once more that this is crucial in the direct calculation of tables of very many vibrational energy transfer rate coefficients, particularly for V–T/R rate coefficients, which require a very long initial separation distance (at least 50 Å) between the colliding molecules. In the next section, we will therefore use the more accurate intramolecular UMN potential in combination with the ILJ intermolecular PES to produce large data sets of such rates, which will be completed through the use of machine learning techniques.

## 5 V–V and V–T/R Complete Data Sets by GPR

The procedure described above was used to calculate V–T/R and V–V coefficients for processes (1), (2), (3), and (4) at various *v* initial quantum numbers with gaps of 1 or 2 quantum numbers, and the source data are reported in the Zenodo repository^51^ as black values. Then GPR models were employed for rate coefficient predictions for the missing quantum numbers *v* in the whole temperature range, and the predicted data are reported^51^ as green values.

The GPR (specified by a covariance/kernel function) uses a set of prior random Gaussian functions to predict the correlation between the Gaussian distributions in two nearby configurations. Specifically, all the computed MQC rate coefficient values have been used to train a GPR model for each Δ*v* within each main process, *i.e.*, we build 11 different GPR models for process (1) (Δ*v* = 1, Δ*v* = 2, and Δ*v* = 3), process (2) (Δ*v* = 1, Δ*v* = 2, and Δ*v* = 3), process (3) (Δ*v* = 1, Δ*v* = 2, and Δ*v* = 3), and process (4) (Δ*v* = 1 and Δ*v* = 2). For each model, the log_10_(*k*) value has been considered as the label (*i.e.*, dependent variable), while the normalized values of both the temperature and the initial quantum number have been used as features (*i.e.*, independent variables). The same settings employed in ref (21) are adopted: a Matern kernel where an extra parameter (*i.e.*, ν, taken here equal to 5/2) in the covariance function is used to specify the smoothness of the resulting function. We employed the GPR approach as implemented in scikit-learn^52^ (the code is freely available at ref (53)), as recently done in ref (13), where we compared the performance of two different machine learning techniques (*i.e.*, GPR and artificial neural network) to predict rate coefficients for the inelastic scattering collisions between N_2_ and H_2_. It is found that, as far as interpolation is concerned, *i.e.*, the *v* values for which rates are predicted fall within the interval of calculated *v*, GPR is able to perform very well in terms of test set mean squared error (MSE) values.^13^

Similar to what has been done in ref (13), the level of confidence of the predicted values is estimated by computing the test set average MSEs, which are reported in Figure S1 in the Supporting Information for different GPR models. For each model, we computed the average MSE value among all possible test sets obtained by removing the MQC rate coefficients of one particular initial vibrational state at a time (apart from the first and last vibrational state). Therefore, for each model, we built *N* – 2 (*N* is the number of initial vibrational states for which the rate coefficients have been computed using the present MQC method) different splits, each one made by considering a single vibrational state as the test set and all of the others as the training set. As an example, to clarify the adopted approach, for process (1) with Δ*v* = 1, the MQC rate coefficients were computed for all temperatures and 27 different initial vibrational states to give 25 splits.

The complete rate coefficient data sets for processes (1–4) within the temperature range of 100–9000 K are then constructed with the help of the above GPR models, starting from training sets made by all the calculated values and are available at ref (51). Specifically, the training set for the process (1) contains rates for the following *v* quantum numbers [1, 2, 3, 5, 7, 8, 10, 12, 15, 18, 20, 22, 25, 27, 28, 30, 32, 35, 38, 40, 42, 45, 47, 50, 52, 54] (with *v* = 1 removed for Δ*v* = 2, 3 and *v* = 2 removed for Δ*v* = 3); for process (2) [1, 2, 3, 6, 8, 10, 12, 15, 18, 21, 24, 27, 30, 33, 35, 37, 38, 39, 40, 41, 42, 43, 44, 45, 46, 48, 50, 52, 54] (with *v* = 1 removed for Δ*v* = 2, 3 and *v* = 2 removed for Δ*v* = 3); for process (3): [1, 2, 3, 4, 5, 6, 8, 10, 12, 14, 16, 18, 20, 22, 24, 27, 30, 33, 35, 38, 40, 43, 45, 47, 50, 51, 52] (with *v* = 1 removed for Δ*v* = 2, 3 and *v* = 2 and 52 removed for Δ*v* = 3); for process (4): [0, 1, 2, 3, 6, 8, 10, 12, 15, 18, 21, 24, 27, 30, 33, 35, 37, 38, 39, 40, 41, 42, 43, 44, 45, 46, 48, 50, 52, 53] (with *v* = 53 removed for Δ*v* = 2).

**Figure 9:**
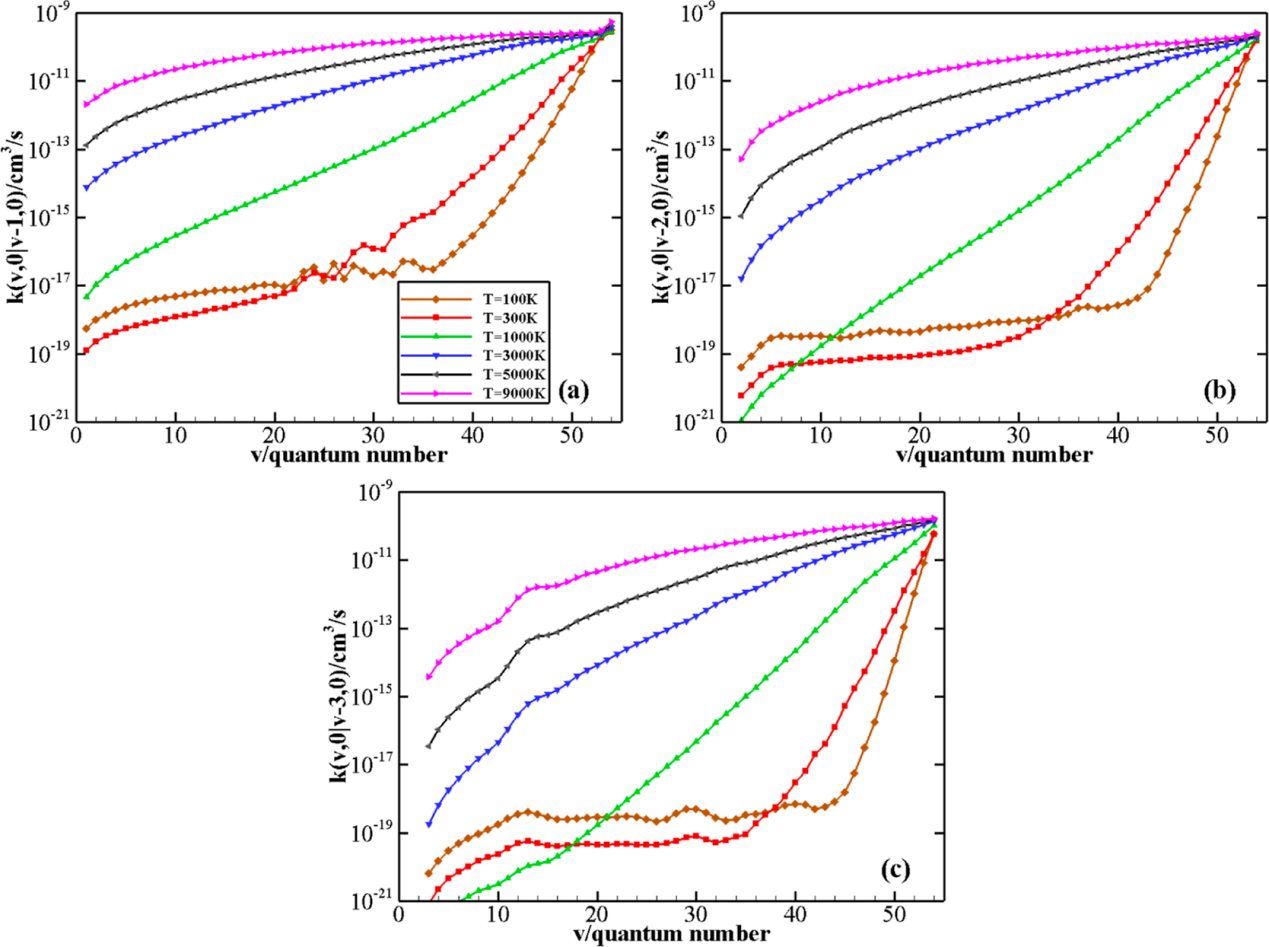
V–T/R rate coefficients for N_2_(*v*) + N_2_(0) → N_2_(*v* – Δ*v*) + N_2_(0) processes, with Δ*v* = 1, 2, 3 (panels a, b, c, respectively) as a function of the vibrational quantum number *v* at different temperature values.

The behavior of rate coefficients for V–T/R processes N_2_(*v*) + N_2_(0) → N_2_(*v* – Δ*v*) + N_2_(0) as a function of the initial quantum number *v* at *T* = 100, 300, 1000, 3000, 5000, and 9000 K is reported in panels (a–c) of Figure 9 for the Δ*v* values (Δ*v* = 1, 2, and 3). The figure shows a significant increase in the rate coefficients with the vibrational excitation of the N_2_(*v*) molecule. The rate of V–T/R processes is extremely low at low temperatures, generally some orders of magnitude smaller than the V–V processes (see below) for the same initial vibrational states. An anti-Arrhenius temperature behavior of the rate coefficients is present in all of the above V–T/R processes for small *v* values. As Δ*v* increases, the value of the vibrational quantum number *v* corresponding to restoring the standard Arrhenius behavior increases, as also reported in ref (8). Besides, a similar behavior of rate coefficients is found for the V–T/R processes N_2_(*v*) + N_2_(1) → N_2_(*v* – Δ*v*) + N_2_(1), as shown in panels (a–c) of Figure S2 in the Supporting Information.

**Figure 10:**
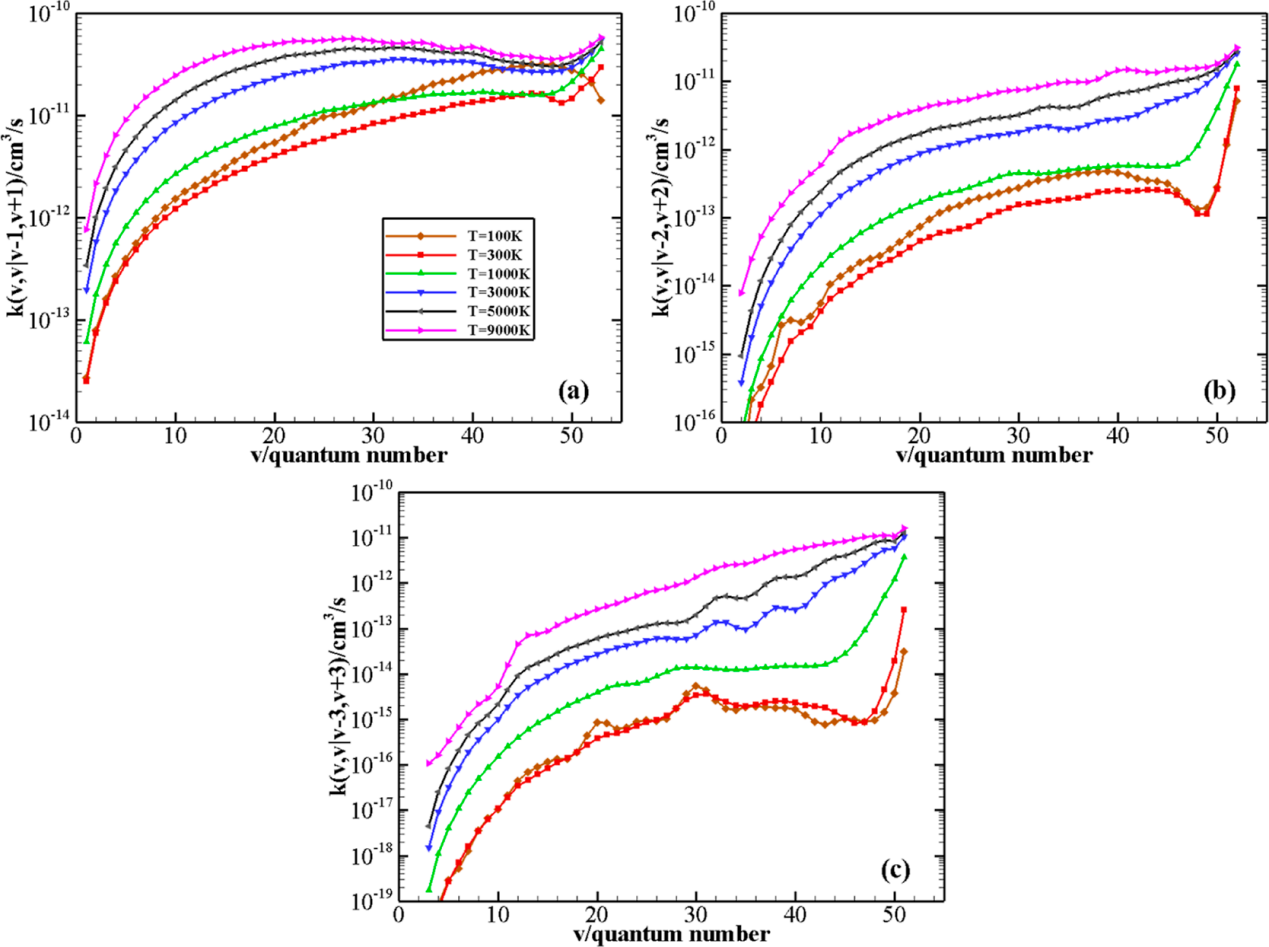
V–V rate coefficients for N_2_(*v*) + N_2_(*v*) → N_2_(*v* – Δ*v*) + N_2_(*v* + Δ*v*) processes, with Δ*v* = 1, 2, and 3 (panels a, b, and c, respectively) as a function of the vibrational quantum number *v* at different temperature values.

The behavior of rate coefficients for the symmetric V–V processes N_2_(*v*) + N_2_(*v*) → N_2_(*v* – Δ*v*) + N_2_(*v* + Δ*v*) as a function of the initial quantum number *v* at *T* = 100, 300, 1000, 3000, 5000, and 9000 K is reported in panels (a–c) of Figure 10 for the Δ*v* values (Δ*v* = 1, 2, and 3). As expected, the efficiency of the symmetric exchange of vibrational quanta of energy decreases as the exchanged number of quanta increases. An anti-Arrhenius behavior of the rate coefficients strongly characterizes the symmetric V–V processes, especially for Δ*v* = 1 and 2 at a very low temperature. A small dip is found around *v* = 47–49 for the Δ*v* = 1 process and at low temperatures for Δ*v* = 2 and 3 processes.

**Figure 11:**
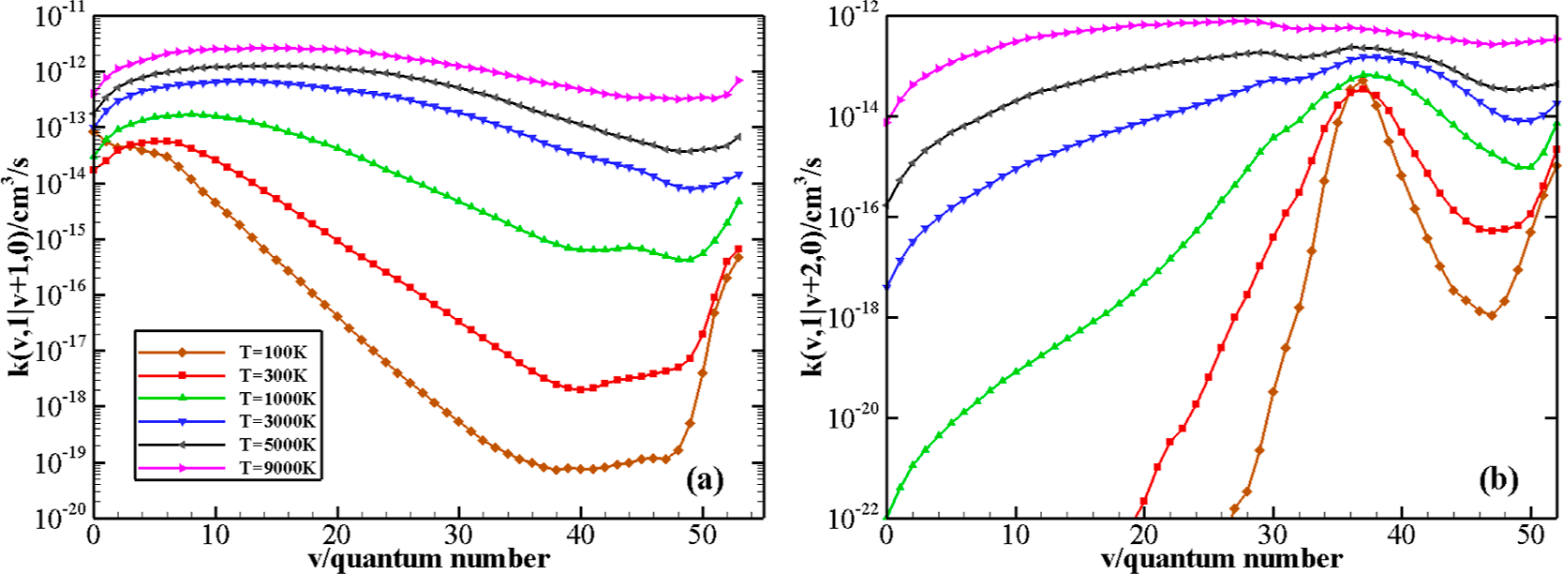
V–V rate coefficients for N_2_(*v*) + N_2_(1) → N_2_(*v* + Δ*v*) + N_2_(0) processes, with Δ*v* = 1, 2 (panels a and b, respectively) as a function of the vibrational quantum number *v* at different temperature values.

The behavior of rate coefficients for quasi-resonant V–V processes N_2_(*v*) + N_2_(1) → N_2_(*v* + Δ*v*) + N_2_(0) as a function of the initial quantum number *v* at *T* = 100, 300, 1000, 3000, 5000, and 9000 K is reported in panels (a,b) of Figure 11 for Δ*v* = 1, 2 values, respectively. It can be noted that the most resonant processes (*i.e.*, those for which Δ*E* approaches zero) are strongly favored, particularly at very low temperatures. This determines a marked decrease of the rate coefficients as *v* increases when Δ*v* = 1 (panel a) and the presence of an anti-Arrhenius behavior is limited to the lowest *v* values (Δ*v* = 1, 2, 3). An increase of rate coefficients, particularly evident at low temperature, is found in proximity to the dissociation limit, perhaps because of the growing exothermicity of the process. The strong rate increase when Δ*E* → 0 is remarkable for Δ*v* = 2 case (panel b): the rate coefficients at low temperature sharply peak for those *v* values corresponding to the smallest Δ*E* (the same figure reporting rates as a function of Δ*E* instead of *v* is given as Figure S3 in Supporting Information). The highest rate is at *v* = 37, whereas the rate dependence upon *v* becomes small at the highest temperature investigated here.

In general, for most of the processes investigated here, high temperature rates show little variation with the *v* value, whereas, at low *T*, specific transitions—mostly corresponding to nearly resonant or very exothermic energy transfer—can be orders of magnitude faster.

## 6 Final Remarks

In this work, we address several important questions involved in the calculation of large data sets of rate coefficients for vibrational energy exchange processes arising from inelastic collisions:

1. We modified our MQC method to include any kind of intramolecular potential in the dynamics, overcoming the restriction of a Morse-like potential of the original program. This allows us to use the method for the calculation of collisional inelastic cross-sections for highly vibrationally excited states up to dissociation limit.
2. We critically evaluated the effect of the intramolecular potential on the calculated rates and on transport properties for the important case of N_2_–N_2_ collisions and showed that the use of *ab initio*-based potential is more reliable than the Morse formulation, which produces significant differences for *v* ≥ 20. However, all the computed rates lie generally within 1 order of magnitude (*i.e.*, even with Morse potential, they are more accurate than those obtained by simple theories or extrapolation techniques from the limited experimental data).
3. We critically evaluated the effect of the intermolecular potential on the calculated rates and on transport properties for the same system and showed that an accurate description of the van der Waals well and of the long-range potential is crucial at low temperature and, for quasi-resonant V–V processes, at all temperatures. PESs that describe very carefully reactive collisions might not be as accurate for the description of inelastic collisions, and their behavior at long-range should be tested before their use for such applications.
4. We used the new code with the best choices for intra- and intermolecular potentials to calculate large data sets of rate coefficients for the V–T/R and V–V processes relevant to determine the vibrational population distribution in non-LTE conditions for N_2_-containing gas and plasma. The data sets were completed by applying a machine learning technique as the GPR. To the best of our knowledge, this is the first time that such complete V–V and V–T/R data sets of explicitly calculated rates (*i.e.*, not obtained by simplified first-order theories or by extrapolation) are produced and made available. It is worth remarking that the rates are obtained by a dynamical first-principle approach that accounts for vibrational quantization and for any kind of quantum effect arising in vibrational motion, which might be particularly relevant at low temperature and does not require any kind of external parameters or binning procedure.

For all of the above reasons, we believe the results to be equally reliable at low and high temperatures and applicable to the modeling of many different environments and of interest to many scientific and engineering communities.

As mentioned in the Introduction, the data for vibrational states nearby the dissociation limit and at very high temperature might be less accurate due to the increased impact of the reactive events on the dynamics. The present MQC method, based on the solution of close coupled equations, does not allow the description of bond breaking and dissociation. Work is in progress on the use of a similar MQC approach,^54^ based on wavepacket propagation, which should allow us to overcome this question (but requiring much longer computing time). Preliminary results for low-lying vibrational states suggest that the differences might not be large. However, inclusion of the reactivity in this framework will lead to a complete and coherent picture of the vibrational energy transfer dynamics.

## Supporting Information Available

Reporting the molecular parameters used in Morse potential; reporting the vibrational energy levels obtained using the LEVEL code based on the UMN, Morse, and PIP-NN N_2_ intramolecular potentials; Average MSE values for the considered GP test sets; V–T/R rate coefficients for N_2_(*v*) + N_2_(1) → N_2_(*v* – Δ*v*) + N_2_(1) processes as a function of vibrational quantum number *v* at different temperature values; and V–V rate coefficients for N_2_(*v*) + N_2_(1) → N_2_(*v* + 2) + N_2_(0) + Δ*E* processes as a function of the energy mismatch Δ*E* at different temperature values (PDF)
