# Supplementary material for: Improved Quantum–Classical Treatment of N2–N2 Inelastic Collisions: Effect of the Potentials and Complete Rate Coefficient Data Sets
Source: J Chem Theory Comput. 2023 Nov 26;19(23):8557–71. doi: 10.1021/acs.jctc.3c01103 (PMC10720385; doi:10.1021/acs.jctc.3c01103)
Supplement: Supplementary file 1 — ct3c01103_si_001.pdf [file ct3c01103_si_001.pdf]

An improved quantum-classical treatment of N_2-N_2 inelastic collisions: effect of the potentials and complete rate coefficient datasets. Electronic Supporting Information

Qizhen Hong,[†] Loriano Storchi,[‡] Quanhua Sun,[†,¶] Massimiliano Bartolomei,[§] Fernando Pirani,[‖] and Cecilia Coletti[*,‡]

†*State Key Laboratory of High Temperature Gas Dynamics, Institute of Mechanics, Chinese Academy of Sciences, 100190 Beijing, China*

‡*Dipartimento di Farmacia, Università G. d'Annunzio Chieti-Pescara, via dei Vestini, 66100 Chieti, Italy*

¶*School of Engineering Science, University of Chinese Academy of Sciences, Beijing 100049, China*

§*Instituto de Física Fundamental - CSIC, C/ Serrano 123, Madrid, Spain*

‖*Dipartimento di Chimica, Biologia e Biotecnologie, Università di Perugia, via Elce di Sotto 8, 06123 Perugia, Italy*

E-mail: ccoletti@unich.it

Table S1: Molecular parameters for N_2 in Morse potential.

r_{eq}	1.0977 Å
β	2.689 Å^{-1}
D_e	9.917 eV

Table S2: Vibrational energy levels obtained using the LEVEL code[1] based on the UMN,[2] Morse, and PIPNN[3] N_2 intramolecular potentials. Units of energies are eV.

v/$\epsilon_{v,j=0}$	UMN	Morse	NN
0	0.1466	0.1457	0.1479
1	0.4380	0.4340	0.4411
2	0.7259	0.7180	0.7307
3	1.0104	0.9977	1.0166
4	1.2913	1.2730	1.2988
5	1.5686	1.5440	1.5771
6	1.8423	1.8107	1.8515
7	2.1124	2.0731	2.1221
8	2.3789	2.3312	2.3887
9	2.6417	2.5850	2.6514
10	2.9008	2.8344	2.9102
11	3.1563	3.0795	3.1651
12	3.4081	3.3203	3.4161
13	3.6562	3.5568	3.6633
14	3.9007	3.7890	3.9067
15	4.1414	4.0168	4.1463
16	4.3785	4.2404	4.3821
17	4.6118	4.4596	4.6142
18	4.8415	4.6745	4.8427
19	5.0674	4.8851	5.0674
20	5.2896	5.0913	5.2885
21	5.5080	5.2933	5.5059
22	5.7226	5.4909	5.7197
23	5.9333	5.6842	5.9298
24	6.1402	5.8732	6.1362
25	6.3432	6.0579	6.3388
26	6.5422	6.2382	6.5377
27	6.7372	6.4143	6.7327
28	6.9281	6.5860	6.9239
29	7.1148	6.7534	7.1110
30	7.2972	6.9165	7.2941
31	7.4753	7.0753	7.4730
32	7.6488	7.2298	7.6475
33	7.8179	7.3799	7.8176
34	7.9821	7.5257	7.9831
35	8.1416	7.6672	8.1438
36	8.2960	7.8044	8.2995

Table S2: Continued.

v/$\epsilon_{v,j=0}$	UMN	Morse	NN
37	8.4453	7.9373	8.4501
38	8.5891	8.0659	8.5952
39	8.7275	8.1901	8.7346
40	8.8600	8.3101	8.8680
41	8.9866	8.4257	8.9951
42	9.1069	8.5370	9.1156
43	9.2206	8.6440	9.2290
44	9.3274	8.7466	9.3349
45	9.4270	8.8450	9.4329
46	9.5190	8.9390	9.5224
47	9.6029	9.0287	9.6028
48	9.6783	9.1141	9.6738
49	9.7446	9.1952	9.7348
50	9.8013	9.2720	9.7854
51	9.8476	9.3445	9.8259
52	9.8828	9.4126	9.8568
53	9.9060	9.4764	9.8794
54	9.9164	9.5359	9.8954
55		9.5911	9.9060
56		9.6420	9.9125
57		9.6886	9.9159
58		9.7308	
59		9.7687	
60		9.8023	
61		9.8316	
62		9.8566	
63		9.8773	
64		9.8936	
65		9.9057	
66		9.9134	
67		9.9168	

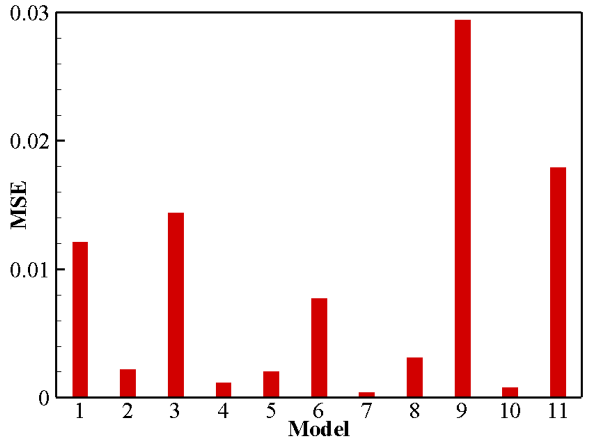

Figure S1: Test set average MSE values of different GPR models: Models 1-3 refer to process (1) with Δv=1, 2, and 3, respectively; Models 4-6 refer to process (2) with Δv=1, 2, and 3, respectively; Models 7-9 refer to process (3) with Δv=1, 2, and 3, respectively; Models 10-11 refer to process (4) with Δv=1 and 2, respectively. In all the cases we built N-2 (i.e., N is the number of initial vibrational states for which the rate coefficients have been computed using the present MQC method) different splits, each one made by considering a single vibrational state as the test set and all the others as the training set.

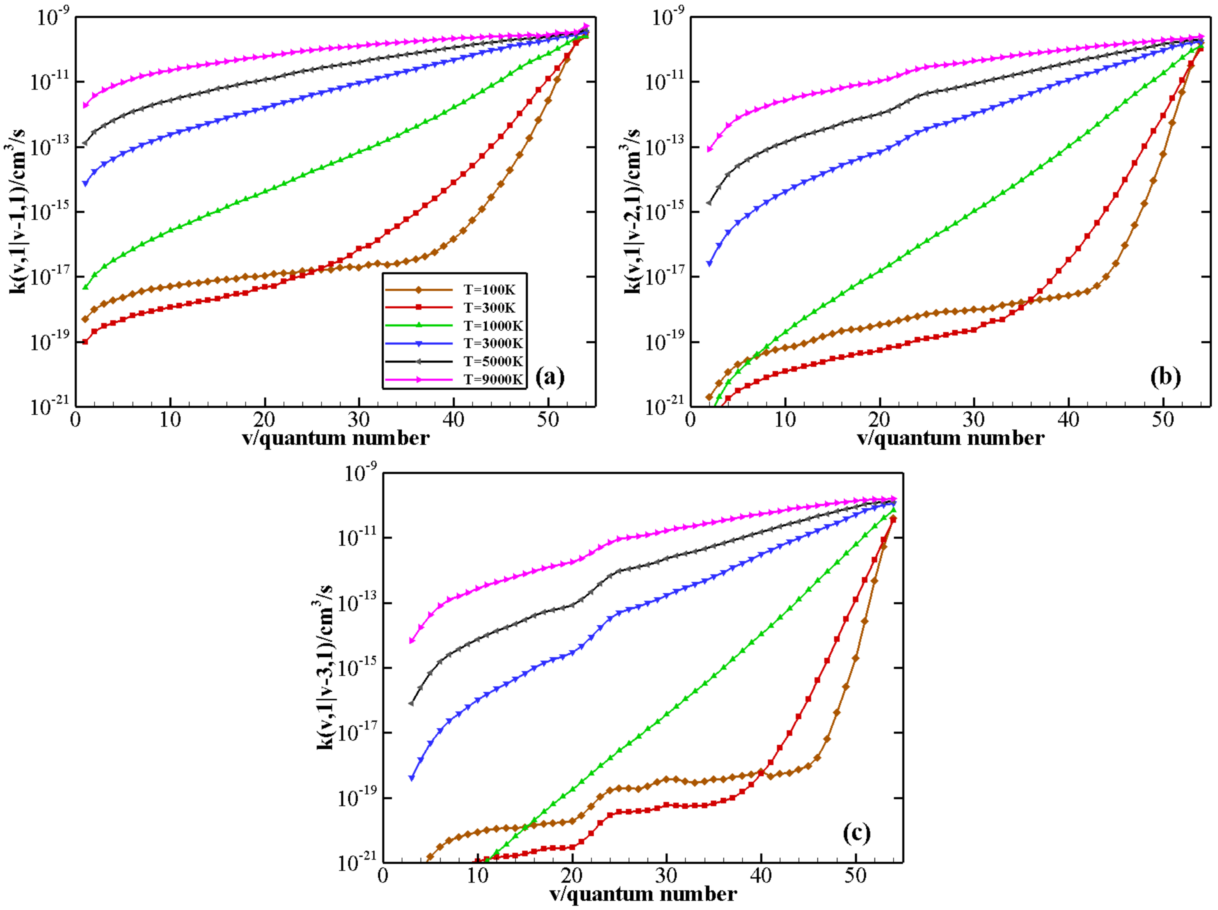

Figure S2: V-T/R rate coefficients for $N_2(v)$+$N_2(1)$$\rightarrow$ $N_2(v-\Delta v)$+$N_2(1)$ processes, with $\Delta v = 1, 2, 3$ (panels a, b, c, respectively), as a function of the vibrational quantum number v at different temperature values.

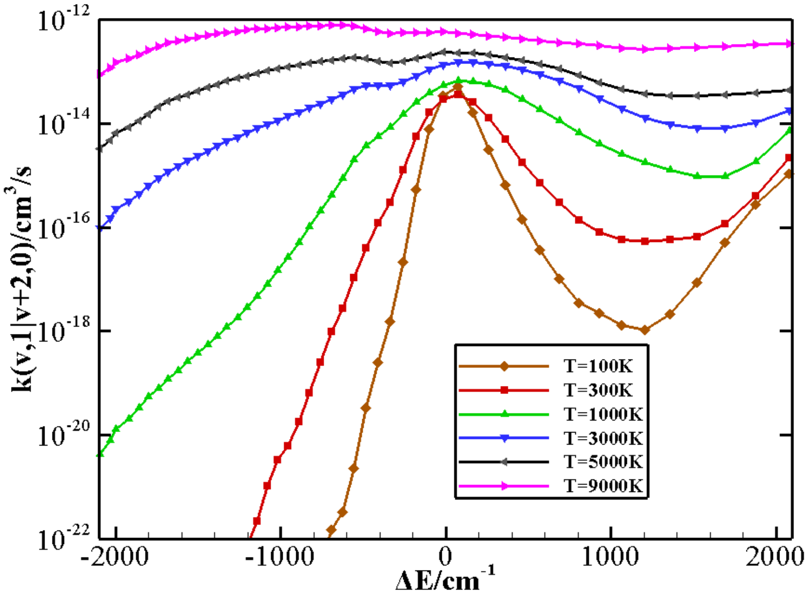

Figure S3: V -V rate coefficients for $N_2(v)$+$N_2(1)$ $\rightarrow$ $N_2(v+2)$+$N_2(0)$ $+\Delta E$ processes as a function of the energy mismatch ΔE at different temperature values.

References

(1) Le Roy, R. J. LEVEL: A computer program for solving the radial Schrödinger equation for bound and quasibound levels. *Journal of Quantitative Spectroscopy and Radiative Transfer* **2017**, *186*, 167–178.

(2) Bender, J. D.; Valentini, P.; Nompelis, I.; Paukku, Y.; Varga, Z.; Truhlar, D. G.; Schwartzentruber, T.; Candler, G. V. An improved potential energy surface and multi-temperature quasiclassical trajectory calculations of $N_2 + N_2$ dissociation reactions. *The Journal of Chemical Physics* **2015**, *143*, 054304.

(3) Li, J.; Varga, Z.; Truhlar, D. G.; Guo, H. Many-Body Permutationally Invariant Polynomial Neural Network Potential Energy Surface for N_4. *Journal of Chemical Theory and Computation* **2020**, *16*, 4822–4832, PMID: 32610014.
